# Learning from Longitudinal Data in Electronic Health Record and Genetic Data to Improve Cardiovascular Event Prediction

**DOI:** 10.1038/s41598-018-36745-x

**Published:** 2019-01-24

**Authors:** Juan Zhao, QiPing Feng, Patrick Wu, Roxana A. Lupu, Russell A. Wilke, Quinn S. Wells, Joshua C. Denny, Wei-Qi Wei

**Affiliations:** 1 Department of Biomedical Informatics, Vanderbilt University Medical Center, Nashville, TN USA; 2 Division of Clinical Pharmacology, Vanderbilt University Medical Center, Nashville, TN USA; 3 Medical Scientist Training Program, Vanderbilt University School of Medicine, Nashville, TN USA; 4 Department of Medicine, University of South Dakota Sanford School of Medicine, Sioux Falls, SD USA; 5 Department of Medicine, Vanderbilt University Medical Center, Nashville, TN USA

## Abstract

Current approaches to predicting a cardiovascular disease (CVD) event rely on conventional risk factors and cross-sectional data. In this study, we applied machine learning and deep learning models to 10-year CVD event prediction by using longitudinal electronic health record (EHR) and genetic data. Our study cohort included 109, 490 individuals. In the first experiment, we extracted aggregated and longitudinal features from EHR. We applied logistic regression, random forests, gradient boosting trees, convolutional neural networks (CNN) and recurrent neural networks with long short-term memory (LSTM) units. In the second experiment, we applied a late-fusion approach to incorporate genetic features. We compared the performance with approaches currently utilized in routine clinical practice – American College of Cardiology and the American Heart Association (ACC/AHA) Pooled Cohort Risk Equation. Our results indicated that incorporating longitudinal feature lead to better event prediction. Combining genetic features through a late-fusion approach can further improve CVD prediction, underscoring the importance of integrating relevant genetic data whenever available.

## Introduction

Cardiovascular disease (CVD) is the leading cause of morbidity and mortality, accounting for one-third of all global deaths^1,2^. Several prediction models have been proposed, including the Framingham risk score^3^, American College of Cardiology/American Heart Association (ACC/AHA) Pooled Cohort Risk Equations^4^, and QRISK2^5^. These models are typically built on a combination of cross-sectional risk factors such as hypertension, diabetes, cholesterol, and smoking status. Although the importance of conventional models cannot be ignored, well-known clinical risk factors for CVD explain only 50–75% of the variance in major adverse cardiovascular events^6^. About 15–20% of patients who experienced myocardial infarction (MI) had only one or two of these risk factors and were not identified as being at “risk” of CVD according to current prediction models^7^. Given the fact that CVD is preventable, and that its first manifestation may be fatal, a new strategy to enhance risk prediction beyond conventional factors is critical for public health.

Electronic health records (EHRs) contain a wealth of detailed clinical information and provide several distinct advantages for clinical research, including cost efficiency, big data scalability, and the ability to analyze data over time. With the wide implementation in the United States, accumulated EHR data has become an important resource for clinical studies^8^. Meanwhile, the recent convergence of two rapidly developing technologies—high-throughput genotyping and deep phenotyping within EHRs – presents an unprecedented opportunity to utilize routine healthcare data and genetic information to accelerate the healthcare. Many institutions and health care systems have been building EHR-linked DNA biobanks to enable such a vision. For example, the BioVU at Vanderbilt University Medical Center (VUMC), as of May 2018, has genotype data of over 50,000 individuals available for research.

Machine learning and deep learning approaches are particularly suited to exploiting such big data for individual outcome prediction, especially when EHRs can be linked to genetic data^9,10^. A recent study from the United Kingdom (UK) applied machine learning to conventional CVD risk factors on a large UK population and improved the prediction accuracy by 4.9%^11^. In this study, we examined: i) the performance of machine learning and deep learning on longitudinal EHR data for the prediction of 10-year CVD event, compared to a gold standard achieved by (ACC/AHA) Pooled Cohort Risk Equations, and ii) the benefits of incorporating extra genetic information.

## Results

### Machine learning and deep learning models with longitudinal EHR data (Experiment I)

This experiment involved EHR data of 109, 490 individuals (9,824 cases and 99, 666 controls, mean age [standard deviation, SD] 47.4 [14.7] years; 64.5% female and 86.3% European). We applied machine learning models using only ACC/AHA features, aggregate and longitudinal EHR features for CVD prediction, respectively. We compared the approaches with a baseline - ACC/AHA equation. We measured the area under a receiver operating characteristic curve (AUROC) and area under a precision recall curve (AUPRC).

**Figure 1:**
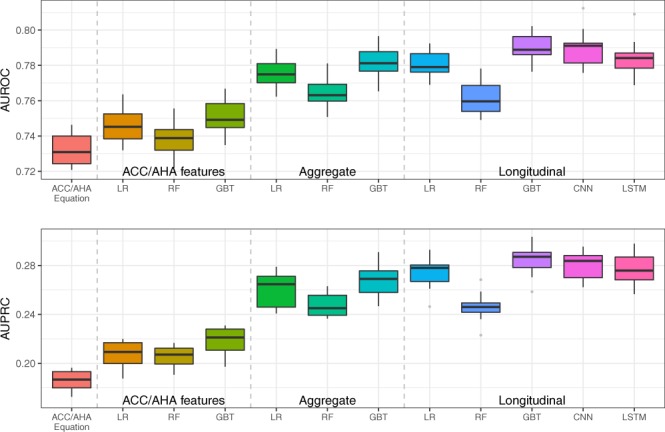
AUROC and AUPRC of gold standard and machine learning/deep learning models for predicting 10-year CVD risk on 10-fold cross validation in Experiment I. The mean values of the AUROC and AUPRC and the standard error are provided in Supplementary Table 1.

The results were reported in Fig. 1 and Supplementary Table 1. Machine learning models all outperformed the baseline ACC/AHA equation. The ACC/AHA equation achieved an average AUROC of 0.732, while machine learning models using only ACC/AHA features obtained an AUROC of 0.738–0.751. By incorporating EHR features, the performance metrics were improved further. Machine learning models using aggregate EHR features achieved an AUROC of 0.765–0.782. By using temporal features, logistic regression (LR), gradient boosting trees (GBT) and deep learning models improved the AUROC to 0.781–0.790. Particularly, GBT and convolutional neural networks (CNN) achieved the highest AUROC of 0.790 (i.e. 7.9% improvement from baseline). For AUPRC, machine learning using temporal features remarkably improved the baseline (i.e., 0.246–0.285 vs. 0.186, a 32.8–44.1% improvement).

#### Feature analysis

We listed the top ten features for each optimized machine learning model (i.e. defined as the selected best model using grid-search on 10-fold cross-validation) in Table 1. For LR, the rank of features was determined by their coefficients (weights). For random forest trees (RF) and GBT, the features were ranked according to the impurity (information gain/entropy) decreasing from a feature. Because CNN and recurrent neural network with long short-term memory (LSTM) units are black box models, estimation of each feature’s contribution to predicting CVD risk remains difficult. We were not able to analyze the feature importance of the deep learning models in this study. We also used the recursive-backwards feature elimination on aggregate features with 5-fold cross-validation to select the top 10 features, shown in Supplementary Table 4.

**Table 1:** Top 10 features for machine learning prediction in descending order of coefficients or feature importance returned by RF and GBT.

LR with aggregate features	RF with aggregate features	GBT with aggregate features	LR with longitudinal features	RF with longitudinal features	GBT with longitudinal features
EHR length	EHR length	Age	EHR length	EHR length	Age
Max LDL-C	Age	EHR length	Age	Age	EHR length
Min Creatinine	Max BMI	SD Creatinine	SD Glucose in 2000	Aspirin in 2006	Smoking
Age	Min BMI	Smoking	SD Creatinine in 2000	Max SBP in 2006	Heart valve disorders in 2006
Max HDL-C	Median BMI	Min BMI	Max HDL-C 2005	Min BMI in 2006	Hypertension in 2006
Max BMI	Max SBP	Heart valve disorders (Phecode 395)	SD Glucose in 2006	Median BMI in 2005	Aspirin in 2006
Max Total Cholesterol	Median SBP	Min Glucose	Median LDL-C in 2006	Median SBP in 2006	Disorders of lipoid metabolism in 2006
Max DBP	SD BMI	Max SBP	Median BMI in 2006	Max BMI in 2006	Clopidogrel in 2006
Median Triglycerides	MIN SBP	Max Triglycerides	Median Total Cholesterol in 2006	Min BMI in 2001	Max SBP in 2006
Min Cholesterol	Max DBP	Aspirin	Heart valve disorders in 2006	Min BMI in 2002	SD Glucose in 2006

LDL-C (LDL Cholesterol); HDL-C (HDL Cholesterol); Systolic Blood Pressure (SBP); Diastolic Blood Pressure (DBP); Body mass index (BMI).

The top features in all machine learning models include some conventional risk factors such as age, blood pressure (BP), and total cholesterol, as well as several new features not included in ACC/AHA features such as body mass index (BMI), creatinine, glucose, and antiplatelet therapy (e.g. Aspirin, and Clopidogrel). Moreover, the maximum, minimum, and SD for laboratory values (e.g. fasting lipid values) and physical measurements (e.g. BMI and BP) contribute more than median values to the models. GBT preferred historically-entered diagnostic codes such as heart valve disorders, lipid disorders, and hypertension) over other features.

For machine learning models with longitudinal features, LR selected laboratory values in the years 2000 and 2006 (e.g. SD Glucose from the observation window). RF chose BMI in multiple years. GBT prioritized the medical conditions obtained from the most recent year before the prediction window (the year 2006).

### Evaluation benefits of additional genetic features (Experiment II)

In this second experiment, we developed a two-stage late-fusion approach to combine genetic and longitudinal EHR features for machine learning models. This is possible at large academic centers where genome-wide data are rapidly moving into EHRs. We compared the approach with ACC/AHA equations, as well as with machine learning models using ACC/AHA features and longitudinal EHR features. The study was conducted using a genotyped cohort of 10,162 individuals (2,452 cases and 7,710 controls with both genotyped data and EHRs from 2000–2016, Supplementary Table 5).

**Figure 2:**
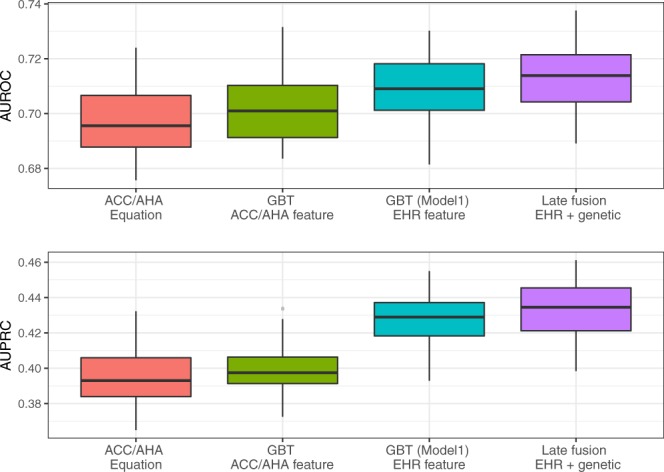
AUROC and AUPRC of gold standard, GBT model on EHR feature and late fusion on EHR and genetic feature for predicting 10-year CVD risk on 50 iterations in Experiment II. The mean values of the AUROC and AUPRC and the standard error are provided in Supplementary Table 2.

The results are shown in Fig. 2 and Supplementary Table 2. GBT using ACC/AHA features generated similar results with ACC/AHA equations. Conversely, GBT using longitudinal EHR features outperformed ACC/AHA equations (AUROC of 0.71 vs. 0.698, AUPRC of 0.427 vs. 0.396). Our innovative late-fusion approach combining additional genetic features then further significantly improved the AUROC and AUPRC by 2.1% and 9.1%.

We listed the top ten features in the pre-trained model with genetic data in Table 2. A single nucleotide polymorphisms (SNP) (rs17465637) was ranked as the second most important feature after age. Many loci that emerged as informative during this approach (*MIA3*, *CXCL12*, and *LPA*) have previously been identified as predictors of early coronary artery disease^12^. We have previously shown that LPA is a strong independent predictor of CVD events on lipid lowering therapy^13^. Chemokines (*CXCL12*) and check point genes (*CDKN2A*) flagged by this approach may alter CVD through their role in angiogenesis. Other loci (*GGCX*) are known to impact response to anticoagulation^14^.

**Table 2:** Top 10 features in pre-trained model 2 with genetic data. Features were ranked according to descending order of absolute value of coefficient effect size.

Features	Reference gene	Coefficient
Age	—	0.747
rs17465637	*MIA3*	−0.334
rs67180937	*MIA3*	0.301
Gender	—	−0.270
EHR length	—	0.180
rs7568458	*GGCX*	0.103
rs4977574	*CDKN2A*	0.095
rs10455872	*LPA*	0.093
rs1412444	*LPA*	0.092
rs501120	*CXCL12*	−0.079

We chose the result from the iteration which generated the closest result from the average AUROC and AUPRC.

## Discussion

This study leveraged a large EHR dataset and EHR-linked biobank in the U.S. to assess the added value of longitudinal data without and with extant genotype. The machine learning models outperformed a traditional clinically-used predictive model for CVD risk prediction (i.e. ACC/AHA equation). Consistent with previous reports^11,15^, we initially observed a benchmark predictability for the ACC/AHA equation of AUROC 0.732 (benchmark for AUPRC was 0.186). When we applied machine learning models using the same ACC/AHA features, they demonstrated better performance (improved AUROC of 0.738–0.751). Because we were interested in whether machine learning models incorporating longitudinal EHR features and genetic features could enhance the prediction, we designed two experiments: i) modeling EHR features with aggregated and temporal features for machine learning and deep learning models, and ii) developing a two-stage late-fusion model to incorporate these longitudinal EHR data with genetic features. The results showed that machine learning models incorporating longitudinal EHR features such as BMI, lab values, and medication history significantly outperformed the baseline approach (improved AUROC 0.761–0.790). Even using a strictly metric AUPRC, the improvement is still significant. We also observed that the late-fusion approach with incorporating genetic data can improve the prediction performance (AUROC of 0.713 vs. 0.698, AUPRC of 0.432 vs. 0.396).

The top features selected in our machine learning models include several conventional risk factors such as age, blood pressure, and total cholesterol. BMI, creatinine, and glucose values (not used in the ACC/AHA equations) were found to be important features, which confirmed previous reports that these features may be independent risk factors for CVD^16–18^. Moreover, the maximum, minimum, and SD of laboratory values (e.g. lipids) and physical measurements (e.g. BMI and BP) provide more discriminating abilities than median values^19^. This finding, that temporal instability in body weight and hemodynamics may be a stronger predictor of risk than cross-sectional estimates of the same parameters, is clinically important.

Longitudinal data more accurately reflect the fluctuation of physiological factors over time. Recently, the STABILITY trial suggested the higher visit-to-visit variation in both systolic and diastolic blood pressure is a strong predictor of CVD^20^. When we narrow our observation window to a one-year slice in time, we captured the longitudinal EHR features year by year. Although the most recent value of some features was often preferred, our machine learning models also have antecedent laboratory tests (e.g. glucose and creatinine in 2000) in their top features when applied to LR. Incorporating these features enhanced the overall performance.

Overall, the study evaluated LR, RF, and GBT using temporal features via two novel deep learning models– CNN and LSTM. CNN is particularly suited to learning local patterns in raw features from input like images, where the intensity of pixels can be combined into higher level features. By comparison, LSTM is designed to learn long/short term dependency of data in a sequence. Both CNN and LSTM outperformed LR and RF but had no measurable advantage over GBT. The reason is that GBT can balance bias and variance to yield better generalization by using a boosting strategy. Although we built the multivariate temporal matrix for each patient, like an 1D image, reduced sequential dependency in the data may not fulfill the advantage of CNN and LSTM. A future study is required to compare GBT, CNN and LSTM on a dataset of more detailed clinical events in a consequent manner.

Lastly, our study also underscores the importance of including genetic variants. CVD has a sizeable hereditary component^3^, and many contributing loci are now being validated through functional studies *in vitro*; the result is a deeper understanding of the biology underlying CVD^21–24^. To date, polygenic risk scoring, a method for summarizing genetic effects for diseases, are being incorporated into clinical practice^25^. However, how to combine genetic variants with other biological and lifestyle factors remain a challenge^26^. We performed a two-stage late-fusion approach and evaluated the predictive power of 204 SNPs with longitudinal EHR data in CVD prediction. Through access to BioVU at the Vanderbilt, the largest single-site biobank in the U.S., we have identified 10,162 individuals with both EHR data and selected SNPs. To enlarge the power of these data, we took advantage of another 34, 926 subjects genotyped cohort in BioVU with selected SNPs available. Our late-fusion approach trained multiple classifiers separately on longitudinal EHR and genetic data, fusing the prediction results across classifiers. Our results demonstrated that genetic features offer benefits to clinical features, resulting in an improved AUROC (i.e. 2.1%) and AUPRC (i.e. 9.1%).

For the model trained with only genetic and demographic features, age remains the strongest predictor for CVD (coefficient 0.747), followed two variants from the *MIA3* gene, gender length of the EHR. This locus has previously been identified as a predictor of early coronary artery disease^12^. Our approach also underscored the importance of the *LPA* gene, a known predictor of CVD events on lipid lowering therapy^13^. Interestingly, in that prior work we reported that variability in the *LPA* gene predicted CVD events independent of circulating lipid levels. Our current observation highlights the importance of this locus in quantifying residual CVD risk even after models have considered lipids.

We acknowledge the limitations that, (1) this study was restricted to data obtained during routine clinical practice, (2) we only used 204 SNPs in our genetic experiment, and (3) that some of the effects of the SNPs may also be modeled directly by phenotypes. Yet, paradoxically, some SNPs for *endophenotypes* are more predictive of CVD events than the endophenotype itself^13^. We believe that, with even denser phenotypic and genetic information available in growing EHR cohorts, prediction would continue to improve. This study confirmed that combining phenotypic and genetic information with robust computational models can improve disease prediction.

## Methods

### Study setting

We conducted the study using data derived from Synthetic Derivative, a de-identified copy of whole EHRs at VUMC. Synthetic Derivative maintains rich and longitudinal EHR data from over 3 million unique individuals, including demographic details, physical measurements, history of diagnosis, prescription drugs, and laboratory test results. As of May 2018, over 50,000 of these individuals have genotype data available.

We focused our analysis on individuals with European or African ancestry. To ensure each individual to have some EHR data, we required an individual to meet the definitions of medical home^27^. We set the baseline date as 01/01/2007 to allow all individuals within the cohort to be followed-up for 10 years. For each individual, we split the EHR into: (i) the observation window (01/01/2000 to 12/31/2006; 7 years) and, (ii) the prediction window (01/01/2007 to 12/31/2016; 10 years). We extracted EHR data in the 7-year observation window (2000–2006) to train a classifier to classify whether the individual would have CVD event in the 10-year prediction window 2007–2016.

We define cases as individuals with ≥1 CVD diagnosis codes (the International Classification of Diseases, Ninth Revision, Clinical Modification [ICD-9-CM]: 411. * and 433. *) within the 10-year prediction window. Controls were individuals without any ICD-9-CM code 411. * or 433. * during the 10-year prediction window.

### Study cohort

The study cohort included patients between the ages of 18 to 78 on 01/01/2000 (beginning of the observation window). Individuals with any CVD diagnosis (ICD-9-CM 411. * or 433. *) prior to the baseline date (i.e. 01/01/2007) were excluded. To reduce chart fragmentation and optimize the density of the longitudinal EHR data, we required each individual to have >=1 visit and >=1 blood pressure measurement during the observation window^28,29^. We excluded inpatient physical or laboratory measures for all individuals.

In total, we identified 109, 490 individuals, including 9,824 cases and 99, 666 controls (mean [SD] age 47.4 [14.7] years; 64.5% female and 86.3% European). The case/control ratio was consistent with a previous report from a large EHR cohort^11^. Among these 109, 490 individuals, 10,162 individuals (2,452 cases and 7,710 controls) had genotype data.

### Data preprocessing and feature extraction

Phenotypic data: we extracted features including demographics, variables in the ACC/AHA equations (e.g. blood pressure measurements), physical measurements (e.g. BMI), and laboratory tests including glucose, triglyceride levels, and creatinine level (as a marker of renal function); such laboratory features have previously been reported relevant to CVD^11^. In addition, we applied chi-square (chi2)^30^, a common feature selection methods to select independent features on EHR data, and identified 40 relevant diagnostic codes and medication codes (Table 3).

**Table 3:** Features included in the machine-learning models.

Feature type	Features	Values
Demographic	Age*	Continuous
	Gender*	Binary
	Race	Categorical
Life styles	Body mass index (BMI)	Summarized data^†^
	Smoking*	Binary
Physical or lab measurements	Systolic blood pressure (SBP)*	Summarized data^†^
	Diastolic blood pressure (DBP)*	Summarized data^†^
	Total Cholesterol (Cholesterol)*	Summarized data^†^
	HDL Cholesterol (HDL-C)*	Summarized data^†^
	LDL Cholesterol (LDL-C)	Summarized data^†^
	Creatinine	Summarized data^†^
	Glucose	Summarized data^†^
	Triglyceride	Summarized data^†^
Diagnosis	Other tests (phecode^33^ 1010)	Binary
	Benign neoplasm of skin (216)	
	Diabetes mellitus* (250)	
	Disorders of lipoid metabolism (272)	
	Other mental disorder, random mental disorder (306)	
	Heart valve disorders (395)	
	Hypertension (401)	
	Cardiomyopathy (425)	
	Congestive heart failure; nonhypertensive (428)	
	Atherosclerosis (440)	
	Acute upper respiratory infections of multiple or unspecified sites (465)	
	Chronic airway obstruction (496)	
	Disorders of menstruation and other abnormal bleeding from female genital tract (626)	
Medication	Warfarin (RXCUI 11289)	Binary
	Aspirin (1191)	
	Atenolol (1202)	
	Amlodipine (17767)	
	Carvedilol (20352)	
	Lisinopril(29046)	
	Adenosine(296)	
	Clopidogrel (32968)	
	Digoxin (3407)	
	Diltiazem (3443)	
	Ramipril (35296)	
	Diuretics (3567)	
	Dobutamine (3616)	
	Simvastatin(36567)	
	Enalapril (3827)	
	Sestamibi (408081)	
	Ethinyl Estradiol (4124)	
	Furosemide (4603)	
	Nitroglycerin (4917)	
	Hydrochlorothiazide(5487)	
	Ibuprofen (5640)	
	Metoprolol (6918)	
	Acellular pertussis vaccine (798302)	
	Atorvastatin(83367)	
	ACE inhibitors (836)	
	Thallium(1311633)	
	Clonidine (2599)	
Genetic	204 SNPs^#^	Categorical
Others	EHR length	Continuous

*Features in ACC/AHA Equations.

^†^Summarized data includes minimum, maximum, median and SD within a time window.

^#^204 SNPs are listed in the Supplementary Data.

We represented a physical measurement or laboratory feature with summarized data, e.g. minimum, maximum, median, and SD. We removed the outliers (>5 SD from the mean) to avoid unintended incorrect measurements (e.g. using lb. instead of kg. for body weight)^31^. If an individual had no such measure within the EHR, we imputed the missing value with the median value of the group with the same age and gender^32^. We also added a dummy variable for each measure to indicate whether the test value was imputed.

For disease phenotypes, we followed a standard approach and grouped relevant ICD-9-CM codes into distinct phecodes^33^. For medications, we collapsed brand names and generic names into groups by their composition (ingredients) and represented the groups using the RxNorm^34^ concepts (RxCUIs) for this variable. For example, ‘Tylenol Caplet, 325 mg oral tablet’ and ‘Tylenol Caplet, 500 mg oral tablet’ were both mapped to ‘Acetaminophen’ (RxCUI 161). We used a binary value to indicate whether or not an individual had each diagnosis or prescription.

For genetic data, we selected 248 SNPs reported to be associated with CVD in two large meta-analyses^23,24^. Among these SNPs, genotype data were available for 204 SNPs in our cohort and were included as features. Each SNP had a value 0, 1, or 2 to represent the count of minor alleles for an individual. Table 3 shows the features used in the machine learning models.

### Experiment

#### Baseline

We chose the ACC/AHA equation for 10-year CVD risk as our baseline. For physical measurements or laboratory features (i.e. SBP/DBP and HDL-c level), we used the most recent values prior to the split date, 01/01/2007.

### Machine learning and deep learning with longitudinal EHR data (Experiment I)

**Figure 3:**
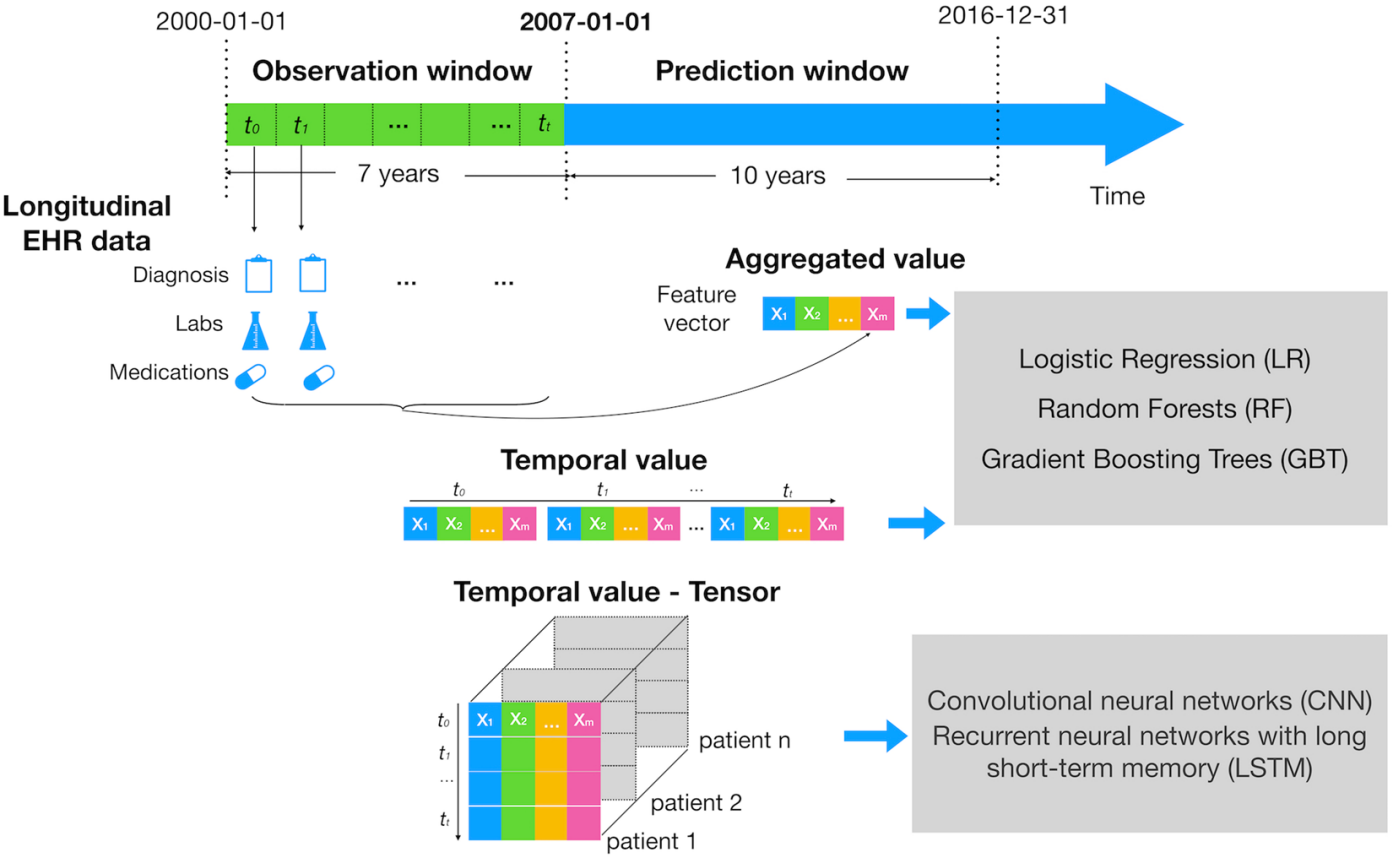
Study design of experiment I. The figure illustrates how we defined the observation and prediction window. It also shows how we modeled longitudinal EHR features: i) We aggregated each feature across the 7-year observation window (e.g. median, max, min and SD of HDL); ii) we extracted each year value of each feature and concatenated the temporal values from all patients into a two-dimensional matrix for a classifier (e.g. LR, RF, GBT); we then constructed a tensor representation on temporal values from all patients for CNN and LSTM.

In this experiment, we used two different ways to model EHR data– extracting aggregate and longitudinal EHR features–for machine learning models (Fig. 3). We compared their performance of 10-year CVD prediction with baseline.

#### Aggregate features

We aggregated features across the 7-year observation window (e.g. median, max, min and SD of HDL from 01/01/2000 to 12/31/2006).

#### Longitudinal features (Multivariate temporal features)

We exploited the temporal information in the longitudinal EHR data by dividing the whole observation window into one-year slice window. Specifically, for physical or laboratory features, we extracted the median, max, min and SD values within one-year slice window. We replaced the missing physical or laboratory measures with the individual’s measurement on the closest date, e.g. using the HDL cholesterol result on 12/20/2005 instead if the individual had no HDL test in 2006. For diagnosis and medication features, we used a binary value to indicate whether or not an individual had each diagnosis or prescription in one-year slice window.

#### Machine learning and deep learning models

Three machine learning models, LR, RF and GBT were used in both aggregate and longitudinal features. Two deep learning models, CNN^35^ and LSTM^36^ were applied to the longitudinal features.

#### Implementation detail

We used CNN and LSTM on longitudinal features and concatenated an auxiliary input of demographic features to feed into a multilayer perceptron (MLP) with two hidden layers. More details can be found in Supplementary Table 3. LR, RF, and GBT were implemented with Python Scikit-Learn 0.19.1 (http://scikit-learn.org/stable/)^37^. The CNN and LSTM models were implemented with Keras 2.1.3 (https://keras.io/) using Tensorflow1.6.1 as the backend. The backward recursive feature elimination was implemented with mlxtend 0.13 (https://rasbt.github.io/mlxtend/).

#### Evaluation

We randomly divided the dataset into a training and a test set with a 90/10 split. We first trained models (LR, RF, GBT) using a grid search with 10-fold stratified cross-validation on the training set to select the best model with the maximum AUROC. Then we tested the selected model on test set. We repeated the above process ten times. For deep learning models, we randomly divided the data into training, validation, and testing sets with a ratio of 8:1:1 and iterated the process ten times. For each iteration, we calculated AUROC and AUPRC^38^ values after applying the model on the test set. We reported the average and SD of both values. To see whether there was a significance difference in the performance, we performed a paired t-test with the level of confidence 0.05.

### Evaluation benefits of additional genetic features (Experiment II)

In this experiment, we examined combining genetic features with demographic and longitudinal EHR data for 10- year CVD prediction. We developed a two-stage late-fusion approach to incorporate EHR and genotyped features. Late-fusion is an effective approach to enhance prediction accuracy by combining the prediction results of multiple models trained separately by a group of features^39^. We trained two sperate models on EHR data and genotyped data, respectively. Then the approach fuses the prediction results by taking the prediction scores as input features and training a fusion model (e.g. LR) for final prediction.

**Figure 4:**
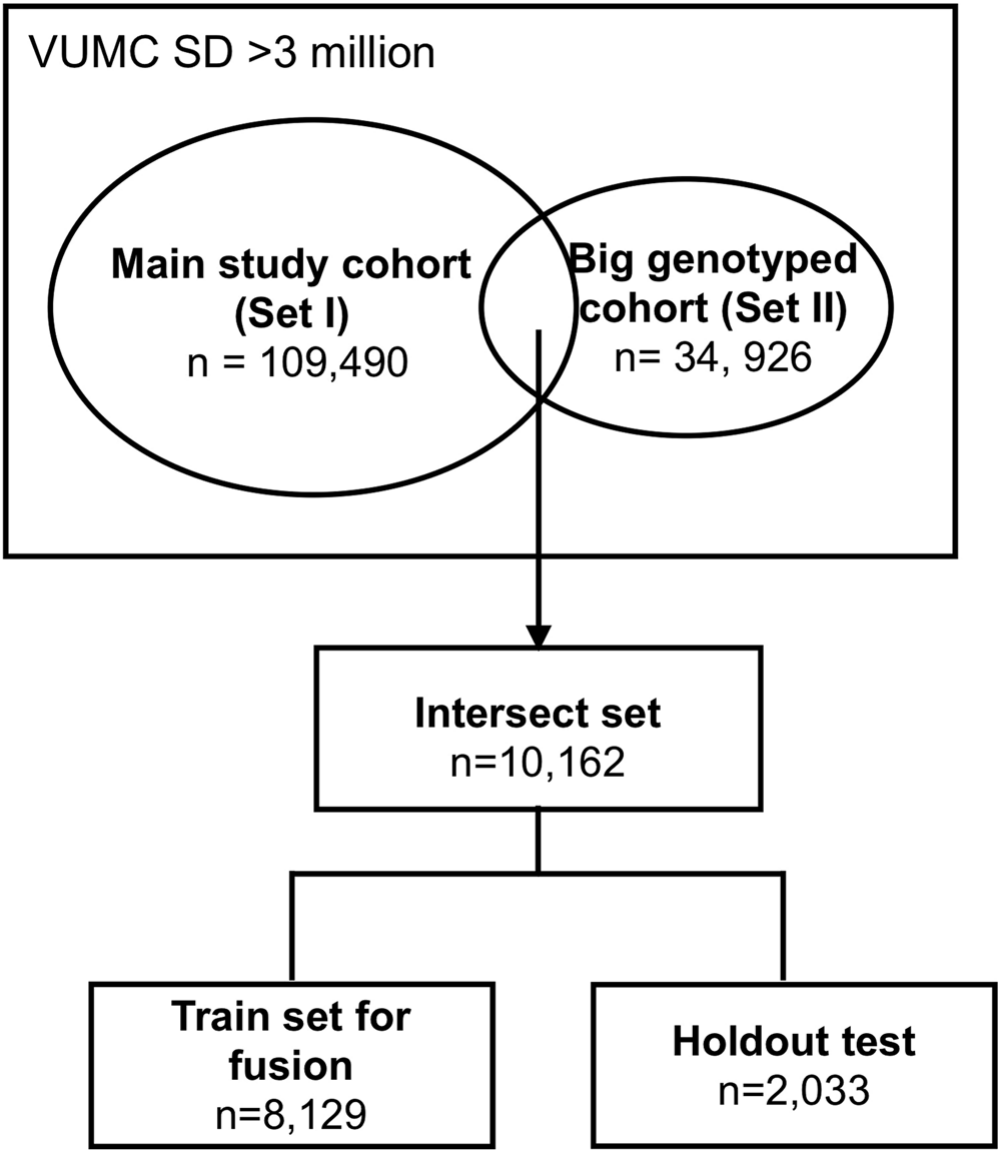
Flowchart of selecting cohort for late-fusion approach.

To enlarge the data power, besides the main study cohort (set I, *n* = 109, 490) used in experiment I, the study utilized another big genotyped cohort (set II) including 34,926 individuals without restricting >1 record of SBP in the observation window (Supplementary Table 5). We used the same criteria (i.e. ≥1 CVD diagnosis codes in the prediction window) to label each individual in the genotyped cohort as a case or control. The set II had 204 SNPs features and basic demographic features (e.g. age, gender, and race). There is an overlap of 10,162 individuals between the two sets (denoted as intersect set), i.e. these individuals have both EHR and genotyped features (Fig. 4). We randomly split the intersect set into a training set (i.e. 8,129 individuals) used for training the fusion model and a holdout test set (i.e. 2, 033 individuals) with an 80/20 split.

**Figure 5:**
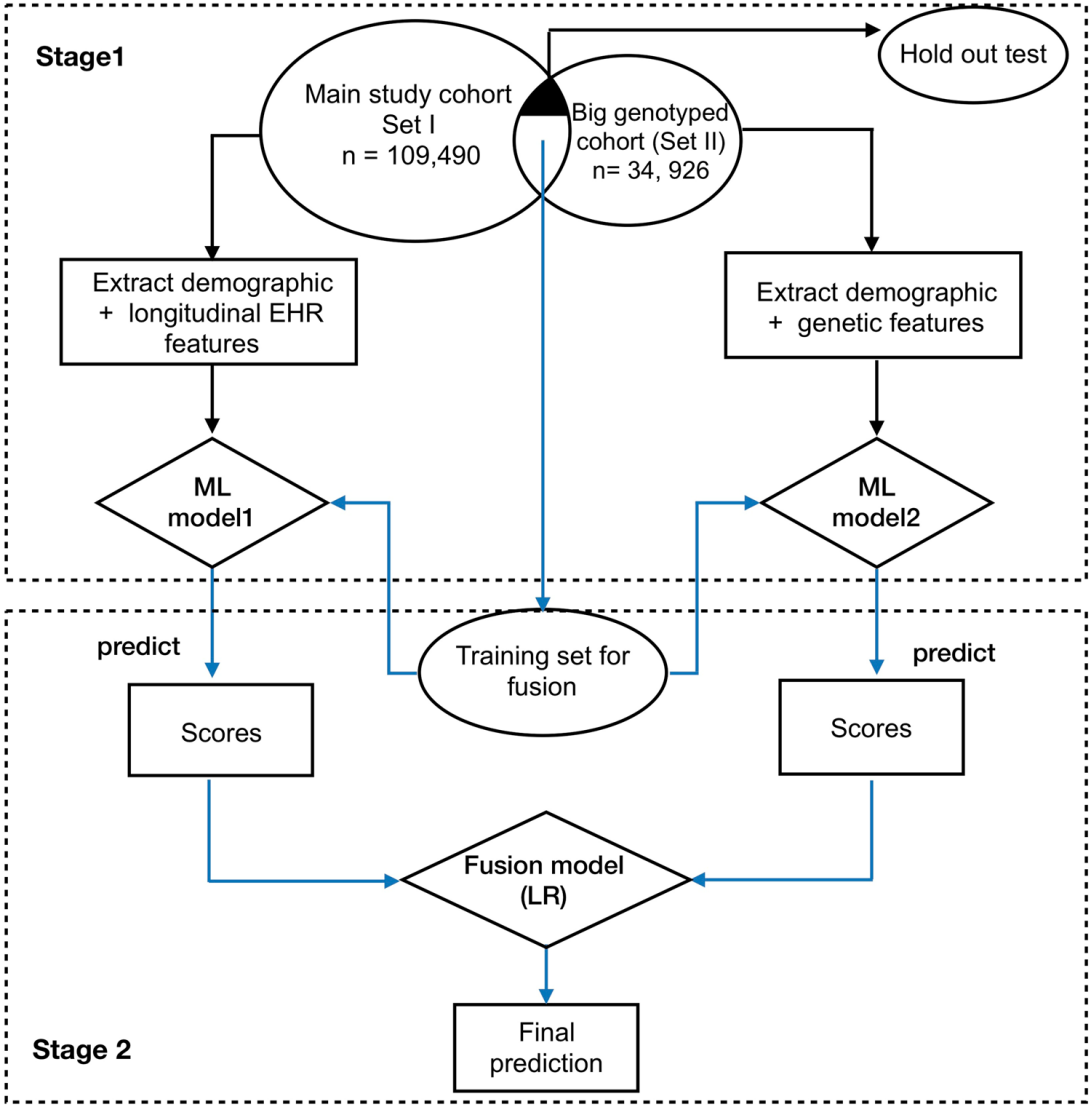
Framework for proposed late fusion approach to combine the genetic features with longitudinal EHR features.

The workflow of the framework is presented in Fig. 5. In the first stage of the framework, we firstly trained a classifier (model1) with longitudinal EHR features on the set I (with holdout test set removed), and trained a another classifier (model 2) using 204 SNPs features on the set II (with holdout test set removed). In the second stage, we applied the model 1 and 2 to the training set from intersect cohort to get prediction scores, which then used as features in fusion model for final prediction.

We used GBT for the model 1 as GBT has good generalizability as an ensemble approach. We used the LR for model 2 and fusion model. To compare the performance, we tested model 1 and fusion model on the holdout test set (2,033 individuals). We performed 5-fold cross-validation and repeated the process ten times. We reported the mean and SD of AUROC and AUPRC.

## Electronic supplementary material

Supplementary Table

Supplementary DATA
