# Supplementary material for: Learning from Longitudinal Data in Electronic Health Record and Genetic Data to Improve Cardiovascular Event Prediction
Source: Sci Rep. 2019 Jan 24;9:717. doi: 10.1038/s41598-018-36745-x (PMC6345960; doi:10.1038/s41598-018-36745-x)
Supplement: Supplementary file 1 — Supplementary Table [file 41598_2018_36745_MOESM1_ESM.docx]

# Learning from Longitudinal Data in Electronic Health Record and Genetic Data to Improve Cardiovascular Event Prediction

Juan Zhao, PhD^1^; QiPing Feng, PhD^2^; Patrick Wu, BS^1,3^; Roxana A. Lupu, MD^4^; Russell A. Wilke, MD^4^; Quinn S. Wells, MD^5^; Joshua C. Denny, MD, MS^1, 5^, Wei-Qi Wei, MD, PhD^1*^

^1^Department of Biomedical Informatics, Vanderbilt University Medical Center, Nashville, TN, USA

^2^Division of Clinical Pharmacology, Vanderbilt University Medical Center, Nashville, TN, USA

^3^Medical Scientist Training Program, Vanderbilt University School of Medicine, Nashville, TN, USA

^4^Department of Medicine, University of South Dakota Sanford School of Medicine, Sioux Falls, SD, USA

^5^Department of Medicine, Vanderbilt University Medical Center, Nashville, TN, USA

* Corresponding author

Email: wei-qi.wei@vumc.org

Department of Biomedical Informatics

Vanderbilt University Medical Center

2525 West End Ave., Suite 1500

Nashville, TN 37203

Tel: (615)343-1956

**Supplementary Table 1. Performance of machine learning and deep learning models predicting 10-year CVD risk. The** + **indicates that the mean is significantly different from the mean of baseline (p < 0.05), when evaluated using the paired *t*-test.** # **indicates that the mean of the model on longitudinal one-year slice window is significantly different from the model with aggregate features.**

| **Method** | **AUROC** | **AUPRC** |
| --- | --- | --- |
| ACC/AHA Equations | 0.732 (± 0.010) | 0.186 (± 0.008) |
| **Machine learning models on recent values of ACC/AHA features (**(i.e., age, gender, race, total Cholesterol, HDL-C, blood pressure, smoking status, diabetes status, hypertension drug treatment) | | |
| Logistic regression (LR) | 0.745 (± 0.009) | 0.207 (± 0.011) |
| Random forest (RF) | 0.738 (±0.009) | 0.205 (± 0.009) |
| Gradient boosting trees (GBT) | 0.751 (± 0.009) | 0.218 (± 0.012) |
| **Machine learning models on aggregate features across seven-year window** | | |
| Logistic regression (LR) | 0.776 (± 0.009) ^+^ | 0.260 (± 0.015) ^+^ |
| Random forest (RF) | 0.765 (± 0.01) ^+^ | 0.247 (± 0.010) ^+^ |
| Gradient boosting trees (GBT) | 0.782 (± 0.009) ^+^ | 0.268 (± 0.014) ^+^ |
| **Machine learning models on longitudinal features within one-year window (temporal)** | | |
| Logistic regression (LR) | 0.781 (± 0.007) ^+#^ | 0.274 (± 0.013)^+ #^ |
| Random forest (RF) | 0.761 (± 0.010) ^+^ | 0.246 (± 0.012) ^+^ |
| Gradient boosting trees (GBT) | 0.790 (± 0.008)^+ #^ | 0.285 (± 0.014)^+ #^ |
| **Deep learning models on longitudinal features within one-year window (temporal)** | | |
| LSTM | 0.784 (± 0.011) ^+^ | 0.276 (± 0.014) ^+^ |
| CNN | 0.790 (± 0.011) ^+^ | 0.280 (± 0.012) ^+^ |

**Supplementary Table 2. Comparison of predicting 10-year CVD risk with genetic features and without genetic features.** + **indicates that the mean is significantly (p < 0.05) different from baseline, and # indicates that the mean is significantly different from GBT using demographic and longitudinal EHR features, when evaluated using the paired *t*-test.**

| **Method** | **AUROC** | **AUPRC** |
| --- | --- | --- |
| ACC/AHA | 0.698 (± 0.012) | 0.396 (± 0.016) |
| **Using ACC/AHA features** | | |
| Gradient boosting trees (GBT) | 0.702 (± 0.012) | 0.399 (± 0.014) |
| **Using demographic and longitudinal EHR features** | | |
| Gradient boosting trees (GBT) | 0.710 (± 0.011) ^+^ | 0.427 (± 0.015) ^+^ |
| **Using demographic, longitudinal EHR and genetic features** | | |
| Fusion approach | 0.713 (± 0.012) ^+#^ | 0.432 (± 0.015) ^+#^ |

**Supplementary Table 3**. Implementation details of machine learning and deep learning models

| **Model** | **Hyper-parameter** |
| --- | --- |
| Logistic regression | *L2*-regularized, tune hypermeters of “*C”: [1,10,100]* |
| Random forest trees | *n_estimators=100,* tune hypermeters of  max_depth: [3, None],  max_features: ['auto', 3, 10],  min_samples_split: [2, 3, 10],  bootstrap: [True, False],  criterion: ["gini", "entropy"] |
| Gradient boosting trees | n_estimators: 100  learning_rate: 0.1  max_depth: 3 |
| CNN | Filters: 48, kernel size :7, maxpooling size: 3  Hidden layer size:2, units: 128, 8  Activation: ReLU  Dropout rate: 0.4, 0.3, learning rate: 0.0005,  Optimizers: Nadam  Max epoch: 80, batch size 128  Batch normalization after each layer |
| LSTM | 41units of LSTM with 1 hidden dense layers.  Hidden layer size: 128  Learning rates: 0.0001  Recurrent dropout: 0.2  Dropout rate: 0.3 |

**Supplementary Table 4 Feature selection using backward recursive feature elimination with 5-fold cross-validation on aggregate features. We used model logistics regression.**

| **Using aggregate features** |
| --- |
| Gender |
| Age |
| EHR length |
| Smoking |
| BMI Missing |
| Max SBP |
| Max BMI |
| SD BMI |
| Disorders of lipoid metabolism( Phecode 272) |
| Heart valve disorders (Phecode 395) |

**Supplementary Table 5 Data characteristics**

| Dataset | Total individuals | Cases | Controls | Mean age (standard deviation) | Female | Europeans |
| --- | --- | --- | --- | --- | --- | --- |
| Main study cohort (Set I) | 109, 490 | 9,824 | 99, 666 | 47.4 (14.7) | 64.5% | 86.3% |
| Big genotyped cohort | 34, 926 | 14, 205 | 20, 721 | 56.0 (14.6) | 52.9% | 88.3% |
| Intersect Cohort | 10, 162 | 2, 452 | 7, 710 | 52.2 (13.4) | 62.6% | 85.5% |

**Supplementary Table 6. Top 10 features for machine learning prediction in descending order of coefficients or feature importance returned by RF and GBT. The numbers in the parentheses indicate the number of occurrences in 10-fold cross-validations. Most features consistently appeared in top 10 features.**

| **LR with aggregate features** | **RF with aggregate features** | **GBT with aggregate features** | **LR with longitudinal features** | **RF with longitudinal features** | **GBT with longitudinal features** |
| --- | --- | --- | --- | --- | --- |
| EHR length (9) | EHR length (10) | Age (10) | EHR length (9) | EHR length (10) | Age (10) |
| Max LDL-C (10) | Age (10) | EHR length (10) | Age (10) | Age (10) | EHR length (10) |
| Min Creatinine (10) | Max BMI (10) | SD Creatinine (10) | SD Glucose in 2000 (9) | Aspirin in 2006 (9) | Smoking (10) |
| Age (10) | Min BMI (10) | Smoking (10) | SD Creatinine in 2000 (1) | Max SBP in 2006 (10) | Heart valve disorders in 2006 (10) |
| Max HDL-C (10) | Median BMI (10) | Min BMI (10) | Max HDL-C 2005 (9) | Min BMI in 2006 (6) | Hypertension in 2006 (10) |
| Max BMI (9) | Max SBP (10) | Heart valve disorders (Phecode 395) (10) | SD Glucose in 2006 (1) | Median BMI in 2005 (5) | Aspirin in 2006 (10) |
| Max Total Cholesterol (9) | Median SBP (10) | Min Glucose (1) | Median LDL-C in 2006 (1) | Median SBP in 2006 (6) | Disorders of lipoid metabolism in 2006 (8) |
| Max DBP (8) | SD BMI (10) | Max SBP (10) | Median BMI in 2006 (3) | Max BMI in 2006 (4) | Clopidogrel in 2006 (10) |
| Median Triglycerides (3) | MIN SBP (10) | Max Triglycerides (1) | Median Total Cholesterol in 2006 (10) | Min BMI in 2001 (1) | Max SBP in 2006 (9) |
| Min Cholesterol (10) | Max DBP (6) | Aspirin (7) | Heart valve disorders in 2006 (1) | Min BMI in 2002 (2) | SD Glucose in 2006 (8) |
